# Prevalence of Nonalcoholic Fatty Liver Disease (NAFLD) in Patients With Type 1 Diabetes Mellitus: A Systematic Review and Meta-Analysis

**DOI:** 10.1210/clinem/dgaa575

**Published:** 2020-08-22

**Authors:** Marieke de Vries, Jan Westerink, Karin H A H Kaasjager, Harold W de Valk

**Affiliations:** Department of Internal Medicine, Diabetology and Vascular Medicine, University Medical Center Utrecht, GA Utrecht, the Netherlands

**Keywords:** nonalcoholic fatty liver disease, NAFLD, NASH, fibrosis, type 1 diabetes mellitus, DM1

## Abstract

**Context:**

Nonalcoholic fatty liver disease (NAFLD) prevalence is high, especially in patients with obesity and type 2 diabetes, and is expected to rise steeply in the coming decades.

**Objective:**

We estimated NAFLD prevalence in patients with type 1 diabetes and explored associated characteristics and outcomes.

**Data Sources:**

We reviewed PubMed and Embase for studies on NAFLD and type 1 diabetes to March 2020. We screened references of included articles.

**Study Selection:**

Two authors independently screened titles/abstracts. One author screened full text articles. NAFLD was defined as described in the individual studies: steatosis and/or fibrosis. Studies not reporting alternative causes of hepatic steatosis or defining NAFLD only as elevated liver enzymes, were excluded. Initially, 919 articles met the selection criteria.

**Data Extraction:**

One researcher performed data extraction and risk of bias assessment using standardized tables.

**Data Synthesis:**

We assessed pooled prevalence rates by meta-analysis using a random-effects model, subsequently exploring heterogeneity by subgroup-, meta-regression-, and sensitivity analysis. Twenty studies between 2009 and 2019 were included (n = 3901). Pooled NAFLD prevalence was 19.3% (95% CI, 12.3%-27.5%), increasing to 22.0% (95% CI, 13.9%-31.2%) in adults only. Pooled prevalence of ultrasound studies was high (27.1%, 95% CI, 18.7%-36.3%) compared to studies using magnetic resonance imaging (8.6%, 95% CI, 2.1%-18.6%), liver biopsy (19.3%, 95% CI, 10.0%-30.7%), or transient elastography (2.3%, 95% CI, 0.6%-4.8%).

**Conclusion:**

NAFLD prevalence in patients with type 1 diabetes is considerable and is highly dependent on the specific diagnostic modality and NAFLD definition used. These data are helpful in directing actions to standardize NAFLD diagnosis, which will help defining contributing mechanisms and outcomes.

Nonalcoholic fatty liver disease (NAFLD) encompasses a spectrum ranging from the relatively benign isolated hepatic steatosis (HS) to the more harmful problems of nonalcoholic steatohepatitis (NASH), hepatic fibrosis, and cirrhosis. NAFLD, by definition, can be diagnosed only in the absence of other causes of liver disease. For diagnosis, many different modalities are used, ranging from laboratory tests, to imaging, to the gold standard, liver biopsy (1-3).

Over the past years, the clinical and economic burden of NAFLD have become apparent and are expected to rise steeply in the coming decades as a consequence of the increased prevalence and incidence of obesity and type 2 diabetes mellitus (4, 5). Global prevalence rates of NAFLD in the general population are estimated at 25% (6). NASH prevalence in the general population is estimated at 1.5% to 6.45%, and 41% within the NASH group develop fibrosis progression (6). Since 2013, NASH cirrhosis is the second leading etiology for liver transplantation (LT) in the United States and in Europe it is anticipated to become the leading indication for LT within the next decade (7, 8). Moreover, mortality due to liver disease is increased in patients with NAFLD (6, 9). The high NAFLD burden is caused not only by these hepatic complications but also by the associated increased cardiovascular morbidity and mortality in patients with NAFLD (6, 9-12).

NAFLD and type 2 diabetes are closely associated phenomena (9, 13-15). NAFLD may be considered as a hepatic manifestation of metabolic syndrome (15-17). In contrast to the knowledge about NAFLD and type 2 diabetes, there are limited and inconsistent data on NAFLD prevalence in patients with type 1 diabetes mellitus (12). Type 1 diabetes and type 2 diabetes show major pathophysiological differences, but share certain similarities as well. Insulin resistance and systemic hyperinsulinemia are seen both in type 1 diabetes and type 2 diabetes (18, 19). Obesity, a well-known NAFLD risk factor clearly related to type 2 diabetes and insulin resistance, is becoming more prevalent in the type 1 diabetes population (6, 20, 21). Taking into account these similarities and adding the generally long lifetime exposure to type 1 diabetes, the spectrum of NAFLD and its long-term sequelae might be clinically relevant in patients with type 1 diabetes as well.

The aim of the present study is to estimate the prevalence of NAFLD in patients with type 1 diabetes. We performed a systematic review and meta-analysis on the prevalence of NAFLD and distribution of its different stages in patients with type 1 diabetes. Moreover, we explored associated characteristics and outcomes of NAFLD in the type 1 diabetes population.

## Materials and Methods

This study was conducted and reported according to the PRISMA (Preferred Reporting Items for Systematic Reviews and Meta-Analyses) statement (22). The research protocol was registered at the PROSPERO database (CRD42019138757).

### Search strategy, selection criteria, and data extraction

We searched PubMed (MEDLINE) and Embase for studies reporting on patients with type 1 diabetes and NAFLD, including synonyms and relevant Medical Subject Headings (MeSH)/Emtree terms. Databases were searched from date of inception to March 2020. We included studies examining patients with type 1 diabetes: children, adolescents, or adults. Studies with or without a control population were both eligible. NAFLD was defined as described and diagnosed in the individual studies. Studies with a diagnosis based solely on abnormal liver tests were excluded. No other restrictions to type of diagnostic method were applied. A description of exclusion of patients with other causes of HS was mandatory. We included cross-sectional and cohort studies published in peer-reviewed journals. Case reports, case series, reviews, posters, abstracts, and descriptive studies were excluded. Language was restricted to English. No limitations to the year of publication were applied. For a detailed description of our search strategy and data extraction plan, we refer to the Supplemental materials (23).

### Risk of bias in individual studies

For risk of bias assessment of the individual studies, we used the risk-of-bias-assessment tool in prevalence studies developed by Hoy et al (24, 25). This was performed at the study level, judging several components of the internal and external validity and composing a summary measure, indicating a low, intermediate, or high risk of bias.

### Statistical analysis

Statistical analysis was performed using SPSS Statistics version 25.0.0.2 and R version 3.5.1, metafor package. For our meta-analysis, we used double-arcsine transformed proportions for better statistical properties (26). Prevalence estimates and 95% CIs were calculated using the DerSimonian-Laird random-effects method. Pooled prevalence rates and 95% CIs were back-transformed into and reported as the original proportion scale. Statistical heterogeneity was tested by using the Cochran Q and *I*^2^ statistic (*I*^2^ values below 25% are considered low, 25% to 50% moderate, 50% to 75% substantial, above 75% high). To give an estimate of the overall NAFLD prevalence in type 1 diabetes patients, all studies were included in the primary analysis, irrespective of precise type 1 diabetes study population, definition of NAFLD, and diagnostic method used. Subsequently, to explore clinical and statistical heterogeneity, we performed subgroup and meta-regression analyses to explore moderators of heterogeneity. We estimated NAFLD prevalence of the following groups: (1) children/adolescents and adults, (2) different diagnostic modalities, (3) NAFLD subclasses. For the meta-regression analysis, we needed at least 5 studies per variable tested. We performed univariate meta-regression analysis on mean body mass index (BMI), mean diabetes duration, and mean glycated hemoglobin (HbA_1c_). We reported parameters of heterogeneity (*I*^2^ and adjusted *R*^2^), and parameters of association (β coefficient, *P* value < .05 considered significant). To alternatively explore the association between NAFLD and BMI, diabetes duration, and HbA_1c_, we performed a meta-analysis, pooling mean differences of these variables between patients without and with NAFLD. To adjust for potential bias within studies, a sensitivity analysis was performed excluding studies with a high risk of bias. The potential for publication bias was explored by a funnel plot and Egger regression test, using double-arcsine transformed proportions.

## Results

**Figure 1.**
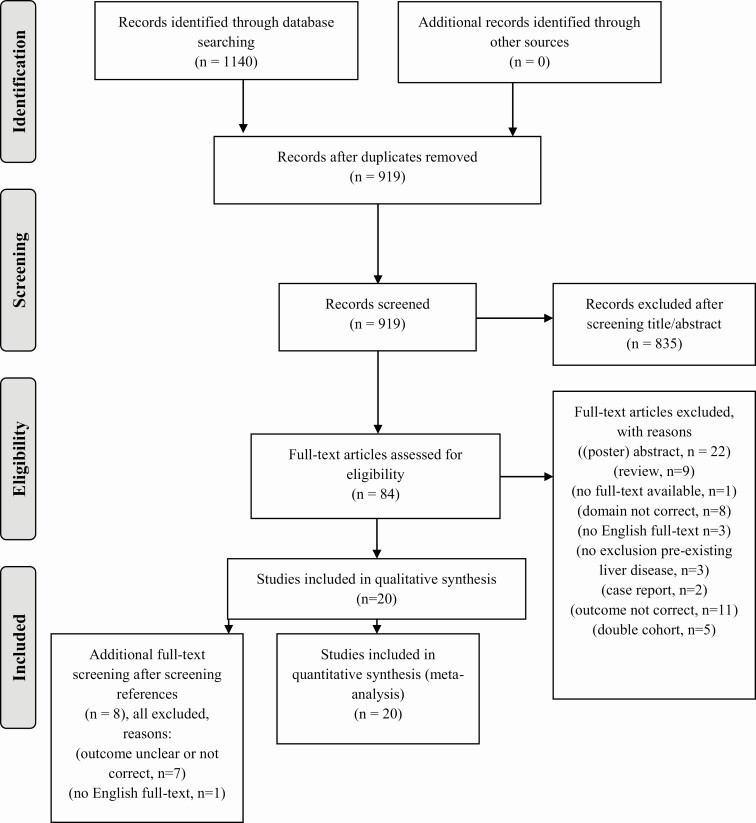
Study flowchart, date of search: March 20, 2020.

Our search yielded 1140 records. After removing duplicates, 919 records remained. After screening titles and abstracts, 835 records were excluded. Subsequently, 84 full-text articles were screened for eligibility, of which 20 studies between 2009 and 2019 were included in the qualitative and quantitative analysis (Fig. 1).

The included studies involved a total of 3901 patients with type 1 diabetes. Table 1 shows the baseline characteristics and reported prevalence rates. The mean age of type 1 diabetes patients in the studies varied from 12.9 to 48.9 years. Three studies evaluated only children and adolescents. Around half of the patients were male. Mean BMI ranged from 19.3 to 28.7 kg/m^2^. Regulation of type 1 diabetes differed between the studies, with mean HbA_1c_ values ranging from 60 to 115 mmol/mol. Mean diabetes duration ranged from 5.9 to 23.4 years. Only a few studies reported on waist circumference and metabolic syndrome, in most studies defined according to the National Cholesterol Education Program Adult Treatment Panel III criteria or World Health Organization criteria (48, 49).

**Table 1.** Baseline characteristics and reported nonalcoholic fatty liver disease prevalence rates of included studies

Study			Population								Diagnostic strategy		Outcome
Author country	Year	Design^*b*^	T1DM pts, No.	Age, y	Male sex, n (%)	BMI, kg/ m^2^	Waist circumference, cm	Metabolic syndrome^*c*^ n (%)	HbA_1c_, mmol/mol %	Diabetes duration, y		Definition of NAFLD	NAFLD prevalence, n (%)
Cipponeri (27) Italy	2019	Cross-sectional, prospective	220	41 (31-49)	88 (40.0)	24.5 (21.9- 26.9)	86 (77-95)	38 (17.3)	61 (53-68)	20.0 (13.0- 30.0)	Ultrasound	Steatosis	57 (25.9)
									7.7 (7.0-8.4)				
Cusi (28) 9 countries^*d*^	2017	Cross-sectional, retrospective	204	39.3 ± 12.2	117 (57.4)	26.5 ± 3.7	–	–	64 ± 13	16.8 ± 11.5	MRI LFC	Steatosis	18 (8.8)
									8.0 ± 1.2				
de Ledinghen (29) France	2012	Cross-sectional, prospective	145	47.8 ± 15.5	80 (55.2)	23.5 ± 3.9	–	35 (24.1)	69 (61-80)	18.0 (12.0- 26.0)	(1) FibroTest	(1) and (2) severe fibrosis (≥ F3)	(1) 4 (2.8)
									8.5 (7.7-9.5)				(2) 3 (2.1)
											(2) FibroScan, M probe		(1) or (2) 6 (4.1)
Elkabanny (30) Egypt	2017	Cross-sectional, prospective	100	13.8 ± 1.9	39 (39.0)	–	–	–	–	–	(1) Ultrasound	(1) Steatosis	(1) 12 (12.0)
													(2) NR
											(2) FibroScan M probe	(2) Fibrosis, mild < F2, significant ≥ F2	
Gaiani (31) Italy	2009	Cross-sectional, prospective	17	–	–	–	–	–	–	–	Ultrasound	Steatosis	4 (24.0)
Harman (32) USA	2014	Cross-sectional, retrospective	57	43.7 ± 17.8	32 (56.1)	25.2 ± 5.4	–	10 (17.5)	–	10.0 (2.1-25.3)	Liver biopsy	Histopathologically confirmed NAFLD	11 (19.3)
Leeds (33) UK	2009	Cross-sectional, prospective	911	42.7 ± NR	515 (56.5)	–	–	–	–	–	(1) ALT preselection, (2) Ultrasound	(1) ALT 2× > 50 IU/L	(1) 17 (1.9)
												(2) Steatosis or fibrosis on US	(2) 9/17 (52.9) HS
													2/17 (11.8) Fib
													➔ 11/17 (64.7)
Li (34) China	2018	Cross-sectional, prospective	39	48 ± 19	19 (48.7)	22.6 ± 4.7	–	–	115 ± 25	–	Ultrasound	Steatosis	6 (15.4)
									12.7 ± 2.3				
Mantovani (35) Italy	2016	Cross-sectional, retrospective	286	43.4 ± 13.7^*a*^	121 (42.3)	24.5 ± 4.1^*a*^	–	–	64 ± 12^*a*^	18.8 ± 12.5^*a*^	Ultrasound	Steatosis	150 (52.4)
									8.0 ± 1.1^*a*^				
Marjot (36) UK	2018	Cross-sectional, prospective	251	40.6 ± 16.7	140 (55.8)	-	–	–	68 ± 18	21.0 ± 15.1	(1) Fib-4 score preselection	(1)Intermediate/high score	(1) 41 (16.3)
									8.4 ± 1.9				
													(2) 2/18 (11.0) Fib
												(2) Significant fibrosis (≥ F2)	
											(2) FibroScan, probe NR		
													➔ 5/251 (1.8)^*e*^
Petit (37) France	2015	Cross-sectional, prospective	128	40.4 (29-51)	65 (50.7)	25.0 ± 4.7	–	–	69 (61-81)	20.0 (11.0- 30.0)	MRI LFC	Steatosis	6 (4.6)
									8.5 (7.7-9.6)				
Regnell (38) Sweden	2015	Cross-sectional, prospective	22	13.5 (9-17)	12 (54.5)	–	–	–	62.5 (46-98)	5.9 (0.0-13.0)	MRI LFC	Steatosis	0 (0.0)
									7.9 (6.4-11.1)				
Serra-Planas (39) Spain	2017	Cross-sectional, prospective	100	39.4 ± 7.8^*a*^	54 (54.0)	24.9 ± 3.2^*a*^	–	–	64 ± 8^*a*^	21.7 ± 2.5^*a*^	Ultrasound	Steatosis	12 (12.0)
									8.0 ± 1.0^*a*^				
Şiraz (40) Turkey	2017	Cross-sectional, prospective	80	12.9 ± 2.4^*a*^	40 (50.0)	20.1 ± 2.3^*a*^	67.6 ± 6.1^*a*^	–	63 ± 7^*a*^	7.9 ± 1.9^*a*^	Ultrasound	Steatosis	8 (10.0)
									7.9 ± 0.7^*a*^				
Sviklāne (41) Latvia	2018	Cross-sectional, prospective	40	44.5 ± 13.2^*a*^	17 (42.5)	28.7 ± 4.3^*a*^	91.6 ± 13.9^*a*^	21 (52.5)	62 ± 27^*a*^	23.4 ± 10.9^*a*^	(1) HSI	(1), (2), and (3) Steatosis	(1) 12 (30.0)
									7.8 ± 0.3^*a*^				
													(2) 14 (35.0)
													(3) 12 (30.0)
											(2) FLI		
											(3) MRI		
Targher^*f*^ (42) Italy	2010	Cross-sectional, retrospective	250	42.5 ± 12.0^*a*^	106 (52.5)	24.8 ± 4.1^*a*^	–	79 (39.1)	66 ± 13^*a*^	18.4 ± 11.6^*a*^	Ultrasound	Steatosis	111 (44.4)
									8.2 ± 1.2^*a*^				
Targher (43) Italy	2012	Cross-sectional, retrospective	343	44.3 ± 14.1^*a*^	156 (45.5)	24.6 ± 4.5^*a*^	88.5 ± 15.1^*a*^	157 (45.8)	78 ± 25^*a*^	15.7 ± 14.7^*a*^	Ultrasound	Steatosis	182 (53.0)
									9.3 ± 2.3^*a*^				
Vendhan (44) India	2014	Cross-sectional, retrospective	736	19.4 ± 11.0^*a*^	384 (52.2)	19.3 ± 4.3^*a*^	69.0 ± 13.2^*a*^	–	88 ± 28^*a*^	7.6 ± 8.3^*a*^	Ultrasound	Steatosis	204 (27.7)
									10.2 ± 2.6^*a*^				
Yoneda (45) Japan	2012	Cross-sectional, prospective	144	48.9 ± 20.4^*a*^	62 (43.1)	22.7 ± 3.6^*a*^	–	–	60 ± 24^*a*^	14.9 ± 13.2^*a*^	Ultrasound	Steatosis	25 (17.4)
									7.6 ± 2.4^*a*^				
Zhang (46) China	2016	Cross-sectional, retrospective	722	46.2 ± 13.1^*a*^	371 (51.4)	21.8 ± 3.8^*a*^	79.1 ± 9.8	274 (38.0)	77 ± 28^*a*^	7.6 ± 4.1^*a*^	Ultrasound	Steatosis	123 (17.0)
									9.2 ± 2.6^*a*^				

Data are mean ± SD, median (IQR), or n (%).

Abbreviations: ALT, alanine aminotransferase; BMI, body mass index; Fib, fibrosis; FLI, fatty liver index; HbA1c, glycated hemoglobin; HS, hepatic steatosis; HSI, hepatic steatosis index; IQR, interquartile range; MRI LFC, magnetic resonance imaging–liver fat content; NAFLD, nonalcoholic fatty liver disease; NR, not reported; pts, patients; T1DM, type 1 diabetes mellitus; UK, United Kingdom; ULN, upper limit of normal; USA, United States of America.

^*a*^Calculated mean ± SD from median (IQR) using formula of Wan (47), and calculated weighted mean from subgroups patients with NAFLD and patients without NAFLD.

^*b*^ *Cross-sectional* means a cross-sectional design for the assessment of NAFLD. *Prospective* means that methods to diagnose NAFLD were performed prospectively. *Retrospective* means that methods to diagnose NAFLD were performed retrospectively.

^*c*^Metabolic syndrome as defined by the different studies.

^*d*^Nine countries: Austria, France, Germany, Italy, Japan (approximately 20% of cohort), Mexico, Poland, Russia, and United States.

^*e*^Prevalence of NAFLD in total T1DM population after extrapolation.

^*f*^Baseline characteristics are described for the patient group without alcoholic fatty liver disease (n = 202). Prevalence rate of NAFLD is calculated from the complete group of patients with T1DM (n = 250).

Different diagnostic strategies were used to evaluate NAFLD: Thirteen studies (65%) used ultrasound, 3 (15%) assessed magnetic resonance imaging liver fat content (MRI LFC), 1 (5%) used a different MRI technique to assess steatosis (comparing brightness of the liver to brightness of the spleen), 2 studies (10%) used a combination of a noninvasive risk score and transient elastography (TE), and 1 (5%) diagnosed NAFLD by liver biopsy. Two studies applied imaging after preselection in the type 1 diabetes population: (1) Leeds et al performed ultrasound only in 17 patients with persistently elevated alanine aminotransferase (ALT) levels higher than 50 IU/L; (2) Marjot et al performed TE only in 11 patients with an intermediate or high risk Fib-4 score and subsequently calculated an extrapolated prevalence rate in their total type 1 diabetes population (33, 36). Most studies defined NAFLD as hepatic steatosis. Two studies looked at significant or severe fibrosis.

**Figure 2.**
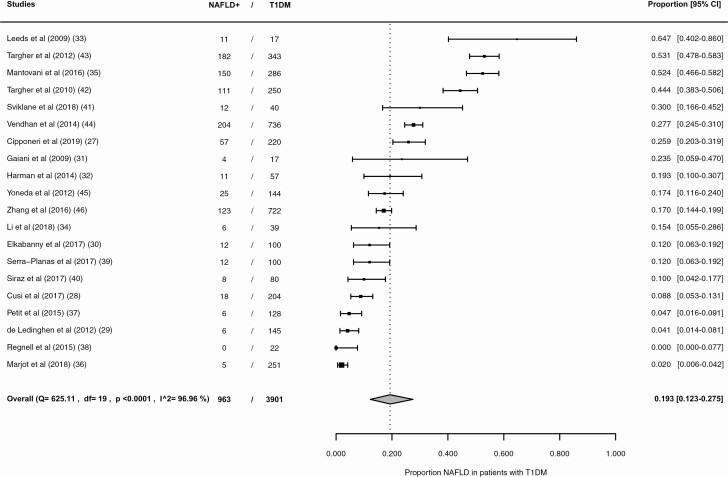
Forest plot overall NAFLD prevalence in patients with type 1 diabetes. NAFLD+, patients with nonalcoholic fatty liver disease; T1DM, type 1 diabetes mellitus.

The results of the individual studies are presented in Fig. 2. The reported prevalence rates range from 0.0% (95% CI, 0.0%-7.7%) to 64.7% (95% CI, 40.2%-86.0%). The pooled NAFLD prevalence is 19.3% (95% CI 12.3–27.5%) (see Fig. 2), with an *I*^2^ statistic showing a high level of heterogeneity of effect sizes of 96.96%.

**Figure 3.**
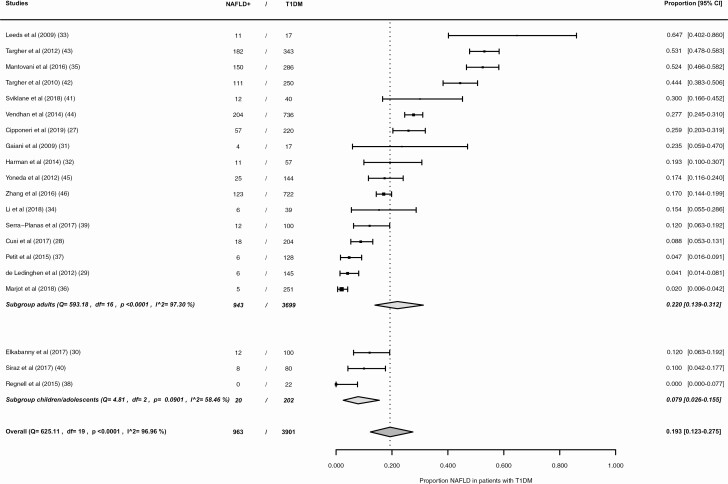
Forest plot NAFLD prevalence in patients with type 1 diabetes, subgroups according to age-group: (1) adults, (2) children/adolescents. NAFLD+, patients with nonalcoholic fatty liver disease; T1DM, type 1 diabetes mellitus.

Of the total type 1 diabetes group, 3699 patients were adults and 202 patients were children/adolescents. Fig. 3 shows the results of this subgroup meta-analysis. The pooled NAFLD prevalence in adults and children/adolescents with type 1 diabetes is 22.0% (95% CI, 13.9%-31.2%) and 7.9% (95% CI, 2.6%-15.5%) respectively. The statistical heterogeneity remained high in the adult group (*I*^2^ = 97.30%). In the subgroup of children/adolescents, it was substantial (*I*^2^ = 58.46%).

**Figure 4.**
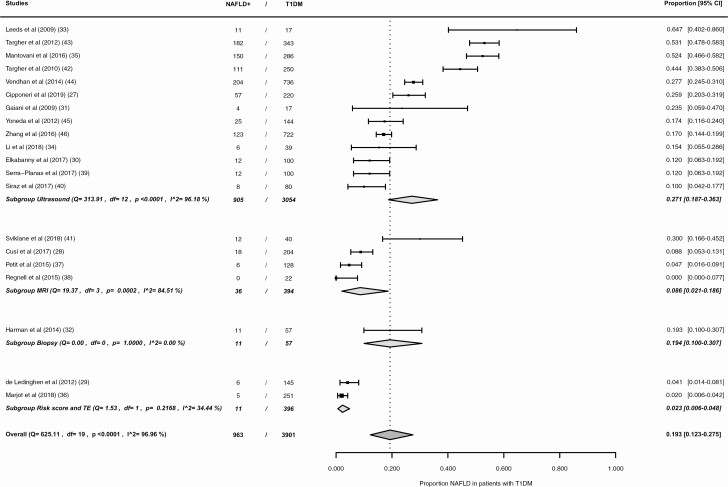
Forest plot NAFLD prevalence in patients with type 1 diabetes, subgroups according to diagnostic modality: (1) ultrasound, (2) MRI, (3) biopsy, (4) combination non-invasive risk score and TE. NAFLD+, patients with nonalcoholic fatty liver disease; T1DM, type 1 diabetes mellitus; MRI, magnetic resonance imaging; TE, transient elastography.

The pooled NAFLD prevalence differed significantly per diagnostic strategy (Fig.4). In ultrasound studies the pooled prevalence is 27.1% (95% CI, 18.7%-36.3%, *I*^2^ = 96.18%). The prevalence is lower in MRI studies, with a pooled prevalence of 8.6% (95% CI, 2.1%-18.6%, *I*^2^ = 84.51%). Using the gold standard, liver biopsy, an NAFLD prevalence of 19.3% (95% CI, 10.0%-30.7%) was found. In the subgroup of studies using a combination of a noninvasive risk score and TE, the pooled NAFLD prevalence is 2.3% (95% CI, 0.6%-4.8%, *I*^2^ = 34.44%).

**Figure 5.**
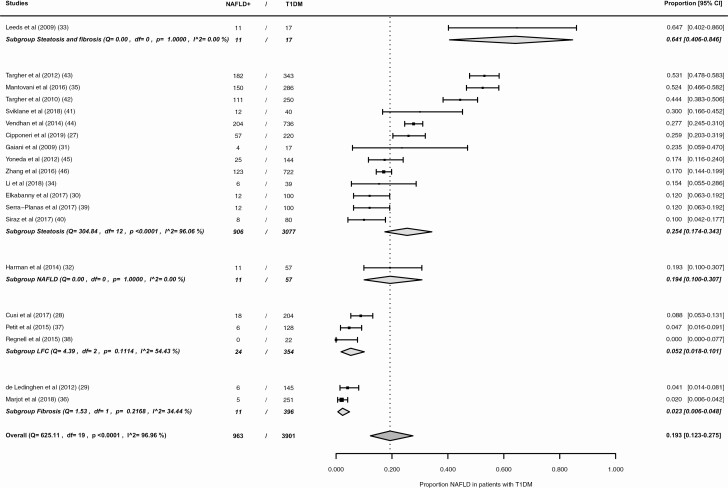
Forest plot NAFLD prevalence in patients with type 1 diabetes, subgroups according to NAFLD definition: (1) steatosis and fibrosis, (2) steatosis, (3) NAFLD, (4) LFC, (5) fibrosis. NAFLD+, patients with nonalcoholic fatty liver disease; T1DM, type 1 diabetes mellitus; LFC, liver fat content.

The pooled NAFLD prevalence differed significantly per NAFLD subset used (Fig. 5). In the majority of studies the reported NAFLD definition is steatosis (n = 13), yielding a pooled prevalence of 25.4% (95% CI, 17.4%-34.3%, *I*^2^ = 96.06%). The pooled prevalence is 5.2% (95% CI, 1.8%-10.1%, *I*^2^ = 54.43%) when steatosis is quantified using MRI, defining steatosis as an LFC of greater than 5.5/6%. Pooling the 2 studies defining NAFLD as significant or advanced fibrosis yields a prevalence rate of 2.3% (95% CI, 0.6%-4.8%, *I*^2^ = 34.44%).

Results of the meta-regression analysis are shown in Figs. S1-S3. None of the tested continuous variables explained any of the heterogeneity of NAFLD prevalence. No significant associations between the explanatory variables and NAFLD were found: mean BMI, β coefficient equal to .011, *P* equal to .650; mean diabetes duration, β coefficient equal to .005, *P* equal to .665; mean HbA_1c_, β coefficient equal to .001, *P* equal to .889. Results of the meta-analysis on mean differences are shown in Figs. S4-S6. Pooled mean differences of BMI, diabetes duration, and HbA_1c_ between patients without and with NAFLD were all significant: BMI –3.0 kg/m^2^ (95% CI, –3.8 to –2.2 kg/m^2^), diabetes duration –5.1 years (95% CI, –9.3 to –0.9 years), HbA_1c_ –2.7 mmol/mol (95% CI, –4.0 to –1.4 mmol/mol).

An overview of risk of bias assessment of the individual studies is given in Table S1. Overall, 8 studies had a high risk of bias, mainly because of a poor external validity in combination with an inconsequent mode of data collection for all participants or a nonoptimal exclusion of other causes of liver disease. Six studies had an intermediate risk of bias due to a moderate external validity. Another 6 studies had a low risk of bias.

Sensitivity analysis, excluding studies with a high individual risk of bias, did not yield any other results (Fig. S7). Although the pooled prevalence estimate increased slightly to 26.1% (95% CI, 17.7%-35.4%), heterogeneity remained high (*I*^2^ = 96.64%). Another sensitivity analysis, excluding the outlier of Leeds et al (33), caused a slight decrease in prevalence, but did not change heterogeneity (pooled prevalence 17.8% (95% CI, 11.0%-25.9%, *I*^2^ = 97.06%). The funnel plot (Fig. S8) shows no asymmetry, and neither Egger regression test was significant, indicating that no publication or related biases were present.

## Discussion

Our systematic review and meta-analysis shows that the overall NAFLD prevalence in patients with type 1 diabetes is substantial at 19.3%, with an even higher prevalence of 22% in adults only. The prevalence estimate is highly dependent on the diagnostic strategy and NAFLD definition used.

Because NAFLD prevalence data in age- and BMI-matched reference groups are scarce, we can only speculate on the prevalence difference between patients with type 1 diabetes and healthy controls. NAFLD prevalence in the general population has been previously estimated at 25% (6). The general population contains considerable numbers of elderly individuals with obesity and/or type 2 diabetes and NAFLD, whereas populations of people with type 1 diabetes are younger and mostly nonobese. The study by Petit et al was the only one in our meta-analysis that had a small adult age- and BMI-matched control group of patients without diabetes; NAFLD prevalence was 13.4% (37). Furthermore, a recent meta-analysis estimated NAFLD prevalence in lean/nonobese individuals at 10% to 16% (50). Therefore, we consider NAFLD prevalence in patients with type 1 diabetes at least comparable to and probably higher than in a matched general population.

NAFLD prevalence in patients with type 1 diabetes is highly dependent on the diagnostic modality used. In general, liver biopsy is considered the gold standard for diagnosing NAFLD. However, in clinical practice only patients with a high index of suspicion undergo liver biopsy. The histologically proven prevalence of 19.3% might therefore be an overestimation of true NAFLD prevalence. Ultrasound may falsely classify patients as having NAFLD because hepatic glycogenosis mimics HS seen on ultrasound (51). MRI LFC has shown to be reliable in quantifying HS compared to liver biopsy and is less prone for confounding by indication and interobserver variability, suggesting this MRI LFC subgroup might reflect the most accurate estimation of pooled NAFLD prevalence (52). The studies using a combination of a noninvasive risk score and TE found low NAFLD prevalence rates because they defined NAFLD as fibrosis, which is much less prevalent than HS in the general and type 2 diabetes population as well (6, 9). Finally, the finding that BMI, diabetes duration (ie, exposure time to insulin resistance and hyperinsulinemia), and diabetes regulation in our analysis were not significant moderators of heterogeneity, supports the idea that the heterogeneity of NAFLD prevalence is substantially explained by diagnostic modality and NAFLD definition.

NASH prevalence was assessed only in the study by Harman et al; a second opinion on 49 liver histology specimens yielded a NASH prevalence of 20.4% (34). On longitudinal follow-up NASH cirrhosis was found in one patient. HCC was reported in 2 patients with type 1 diabetes, but underlying liver disease in these patients was not specified (34). None of the studies reported on histologically confirmed NAFLD fibrosis in patients with type 1 diabetes patients. TE-defined NAFLD fibrosis prevalence was low, but this might be an underestimation of true NAFLD fibrosis prevalence. In the TE studies a substantial number of patients was excluded from further analysis because preselection was applied, no valid FibroScan measurement was obtained, only M probe was available, or patients were not willing to undergo TE. Currently, there are no longitudinal data on decompensated liver cirrhosis and LT in patients with type 1 diabetes and NAFLD, nor on liver-specific, cardiovascular, and all-cause mortality, precluding any detailed discussion on these long-term sequelae.

Our study has several strengths. We performed an extensive systematic literature search and meta-analysis including the best available data on NAFLD prevalence in patients with type 1 diabetes. To limit the amount of bias, we excluded studies using only laboratory tests for diagnosing NAFLD. These have shown to be nonreliable for diagnosing NAFLD because the majority of patients with NAFLD have normal aspartate aminotransferase and ALT values (53). Also, we performed a risk of bias assessment by using a tool especially developed for prevalence studies. Another strength is that we have shown the overall prevalence in the whole type 1 diabetes population as well as in children/adolescents and adults separately.

The most important limitation of this meta-analysis is that the study populations, diagnostic method, and outcome definitions differed so much across studies. We tried to explain heterogeneity in NAFLD prevalence to the best of our ability. Still, because of the small number of studies, performed in selected patient populations, we could perform only exploratory subgroup and meta-regression analysis, and we were not able to draw any unambiguous conclusions on associations between continuous variables and NAFLD. Moreover, we could not elaborate on characteristics associated with the more severe NAFLD stages. Finally, the studies in this review did not allow any conclusion on long-term vascular or hepatic consequences of NAFLD in patients with type 1 diabetes.

Speculating on the future NAFLD burden in patients with type 1 diabetes, we believe that the prevalence of NAFLD in patients with type 1 diabetes may increase considerably in the coming decades. The growing obesity prevalence in the type 1 diabetes population will come with an increase in insulin resistance, an important clinical factor associated with NAFLD progression (20, 54). Patients will combine features of type 1 diabetes with those of type 2 diabetes, with increased prevalence of dyslipidemia and hypertension. This is labeled as “double diabetes,” and is associated with an increased cardiovascular risk (55). Moreover, patients with type 1 diabetes usually have a lifetime exposure to diabetes mellitus. These developments will presumably lead to an increased risk for cardiovascular events as well as advanced NAFLD in patients with type 1 diabetes (9).

In conclusion, our meta-analysis shows that NAFLD prevalence in patients with type 1 diabetes is considerable, and is at least comparable to and probably higher than in an adequately matched sample of the general population. The prevalence estimate is highly dependent on the specific diagnostic modality and NAFLD definition used. From this meta-analysis, we cannot draw any conclusions on the risk of progression of disease to more harmful NAFLD stages and long-term NAFLD-related outcomes. In addition to the well-known NAFLD risk groups of patients with obesity and type 2 diabetes, patients with type 1 diabetes should—with their changing phenotype—be considered a group at risk for NAFLD.

## Data Availability

The data sets generated during and/or analyzed during the present study are not publicly available but are available from the corresponding author on reasonable request.

## Abbreviations

ALT: alanine aminotransferase
BMI: body mass index
HbA_1c_: glycated hemoglobin
HS: hepatic steatosis
LFC: liver fat content
LT: liver transplantation
MRI: magnetic resonance imaging
NAFLD: nonalcoholic fatty liver disease
NASH: nonalcoholic steatohepatitis
TE: transient elastography

## Additional Information

***Disclosure Summary:*** The authors have nothing to disclose.
